# Up-regulated miR-106b inhibits ox-LDL-induced endothelial cell apoptosis in atherosclerosis

**DOI:** 10.1590/1414-431X20198960

**Published:** 2020-03-02

**Authors:** Yunqing Zhang, Li Wang, Jie Xu, Xiaomei Kong, Lin Zou

**Affiliations:** 1 Department of Cardiology, Zuanshiwan Branch of The Second Affiliated Hospital of Dalian Medical University, Dalian, Liaoning, China; 2 Department III of Cardiology, The Central Hospital of Dalian, Dalian, Liaoning, China; 3 Department of Endocrinology, Zuanshiwan Branch of The Second Affiliated Hospital of Dalian Medical University, Dalian, Liaoning, China

**Keywords:** Atherosclerosis, ox-LDL, miR-106b, PTEN, P13K/AKT, Endothelial cell apoptosis

## Abstract

This research aimed to explore the molecular mechanism of microRNA (miR)-106b in cell apoptosis of atherosclerosis (AS). Human aortic endothelial cells (HAECs) were divided into control group, oxidized-low-density lipoproteins (ox-LDL) group, miR-106b NC+ox-LDL group, miR-106b mimics+ox-LDL group, miR-106b mimics+PTEN+ox-LDL group, and miR-106b mimics+empty+ox-LDL group. Real-time fluorescence quantitative polymerase chain reaction, cholecystokinin, TdT-mediated biotinylated nick end-labeling assay, luciferase reporter gene assay, and flow cytometry analysis were performed to determine the morphology, proliferation, and apoptosis in HSECs. Moreover, the levels of phosphatase and tensin homolog deleted on chromosome 10 (PTEN), Bcl-2, p-P13K, and p-AKT in HAECs were detected by western blot. MiR-106b was down-regulated in ox-LDL-induced HAECs. PTEN was the target gene of miR-106b-5p. Overexpression of PTEN inhibited the anti-apoptotic effect of miR-106b. Compared with the control group, the proportion and number of HAECs apoptosis and Bax, caspase-3, and caspase-9 expression in ox-LDL and miR-106b mimics+PTEN+ox-LDL groups were significantly increased (all P<0.05). Moreover, the activity of HAECs and Bcl-2 were decreased significantly (all P<0.05). Overexpression of miR-106b in ox-LDL-induced AS inhibited endothelial cell apoptosis. Furthermore, miR-106b might activate the PI3K/AKT pathway by down-regulating the expression of PTEN in ox-LDL-induced HAECs.

## Introduction

Atherosclerosis (AS) is a chronic inflammatory process of the arterial wall caused by hyperlipidemia (1). There are two prominent hallmarks of AS pathogenesis including the accumulation of cholesterol in the endothelial lining of arteries carried by low-density lipoproteins (LDL) and chronic inflammation due to a high ratio of prooxidants to antioxidants (2,3). As a systemic disease, AS and its accompanying clinical complications are important factors in long-term mortality and morbidity worldwide (4,5). Although various strategies such as drug therapy and surgery have been used for the clinical treatment of AS, the outcome of AS treatment is still not optimal due to the lack of a deep understanding of the pathological mechanism of AS (6,7).

MicroRNAs (miRNAs) act as significant regulators in the pathophysiology of AS (8). As a member of miRNAs, the miR-106b family is associated with the level of genes regulating the cell cycle, which promotes cancer cell proliferation by shortening cell cycle progression (9). The differential expression of miR-106b has been proven to take part in the development of various diseases such as prostate cancer, lung cancer, and gastric cancer (10–12). A previous study shows that the abnormal expression of miR-106b is closely related with AS progression (13).

The biological function of miR-106b in disease can be via certain pathways (14). Yan et al. (15) showed that drugs could inhibit inflammation and promote the stability of AS plaques by regulating the PI3K/Akt pathway. Phosphatase and tensin homolog deleted on chromosome 10 (PTEN) is a viable target for the prevention of apoptosis in vascular endothelial cells (16,17). A previous study indicated that miR-106b promotes pituitary tumor cell proliferation and invasion through PI3K/AKT signaling pathway by targeting PTEN (18). Shi et al. (19) reported that miR-106b-5p participates in the PI3K/AKT pathway by regulating PTEN, thereby promoting stem cell-like properties in liver cancer cells. Furthermore, human aortic endothelial cells (HAECs) are commonly used for the pathological study of AS since cytokine secretion by HAECs is related to the degree of AS (20). Previous study indicates that miR-21 suppresses oxidized-LDL (ox-LDL)-induced HAECs injuries in AS (21). Although the function of miRNA-106b has been reported in the development of various diseases, the relationship between miR-106b and AS is still not clear.

In this study, the mechanism of miR-106b in AS was explored based on ox-LDL-treated HAECs. Biological function of miR-106b in ox-LDL-treated HAECs progression was investigated. Our findings suggested that miR-106b exerted a pro-proliferation role in ox-LDL-treated HAECs and could activate the PI3K/AKT pathway.

## Material and Methods

### Cell culture and transfection

HAECs were cultured in endothelial cell culture medium containing 1% endothelial growth factor and 5% fetal bovine serum (FBS) at 37°C (5% CO_2_). HAECs were transfected with miR-106b NC, miR-106b mimics, PTEN expression plasmid, or empty plasmid (Shanghai Gemma Gene, China) by Lipofectamine 2000 for 24 h, followed by ox-LDL (50 μg/mL, Beijing Xiesheng Biotechnology Co., Ltd., China) treatment for 24 h. Then, all cells were divided into ox-LDL group, miR-106b NC+ox-LDL group, miR-106b mimics+ox-LDL group, miR-106b mimics+PTEN+ox-LDL group, and miR-106b mimics+empty+ox-LDL group. Cells without ox-LDL treatment were considered as the control group.

### CCK-8 assay

Cell activity was detected by CCK-8 assay. Briefly, HAECs were seeded on a 96-well plate (1×10^4^ cells/well) with a concentration of 10 μL/well CCK-8 solution (Engreen Biosystem, China). Plates were incubated at 37°C for 2 h, and absorbance at 450 nm was recorded by a microplate reader (Bio-Rad, USA).

### Flow cytometry assay

Flow cytometry was used for HAECs apoptosis detection. Briefly, after re-suspended with 100 μL PBS, cells from each group were suspended with 5 μL FITC-AnnexinV (1 μg/mL) and 5 μL PI (1 μg/mL). Then, all cells were quantitatively detected by FACScan flow cytometry (BD Biosciences, USA) with CellQuest software (BD Biosciences).

### TdT-mediated biotinylated nick end-labeling (TUNEL) assay

TUNEL assay was performed to detect the HAECs cell apoptosis from each group. According to the TUNEL kit (Beyotime Biotechnology, China) instructions, the HAECs were fixed with 4% formaldehyde and then permeated with 0.1% Triton X-100. Next, the cells were cultured with the mixture of TUNEL reaction at 37°C for 1 h. Finally, HAECs were observed under a laser confocal microscope (FV300, Olympus, Japan).

### RT-qPCR

The expression of miR-106b was detected using RT-qPCR (ABI7500 (Thermo Fisher Scientific, USA). Total RNAs were extracted from cells using TRizol reagent (Invitrogen, USA), and reverse transcription was performed using Takara reverse transcription kit (Japan). The amplification primer sequence of each gene is listed in Table 1. In addition, U6 was the internal reference. The data were analyzed by the 2^-ΔΔCt^ method (22).

**Table 1.** Primer sequences.

Primer	Primer sequences
U6-F	CTCGCTTCGGCAGCACATATACT
U6-R	ACGCTTCACGAATTTGCGTGTC
miR-106b-F	CTGGAGTAAAGTGCTGACAGTG
miR-106b-R	GTGCAGGGTCCGAGGT
PTEN-F	CAAGATGATGTTTGAAACTATTCCAATG
PTEN-R	CCTTTAGCTGGCAGACCACAA
GAPDH-F	GGAAGGTGAAGGTCGGAGTCA
GAPDH-R	GTCATTGATGGCAACAATATCCACT

### Western blot assay

The expression of proteins in HAECs was detected by western blot. Total protein of HAECs from different groups were extracted by RIPA lysis buffer (Beyotime Biotechnology) containing protease and phosphatase inhibitors (complete ULTRA Tablets, Roche, USA). After centrifugation at 12,000 *g* for 5 min at 4°C, proteins were separated by 10% SDS-polyacrylamide gel electrophoresis and transferred to a polyvinylidenefluoride membrane (Roche). The membrane was then blocked with 50 g/L skim milk for 12 h at 4°C, and incubated with primary antibodies: GAPDH, β-actin, PTEN, Bcl-2, Bax, caspase-3, caspase-9, p-P13K, P13K, p-AKT, and AKT (1:1000; Cell Signaling Technology, USA) overnight at 4°C. Then, the membrane was incubated with secondary antibody (HRP-conjugated anti-mouse IgG, 1:2000, Beijing Boaosen Biotechnology Co., Ltd., China) for 2 h at 37°C. Protein bands were visualized using Epson photo 1650 (Seiko Epson Corporation, Japan). Finally, β-actin was used as an internal reference (1:1000, sc-517582, Santa Cruz Biotechnology, USA), and the relative gray value was analyzed using Quantity One scanning software (Bio-Rad Laboratories, USA).

### Luciferase reporter gene assay

The target sites for miR-106b-5p were determined by Target Scan (http://www.targetscan.org), and the mutant and wild sequences were designed according to the predicted results. The miR-106b-5p mutant sequence and the wild sequence fragment were cloned and bound to the PGL-3 vector. Then, the 293T cells (ATCC) were seeded onto 24-well plates (5×10^5^ cells/well) and co-transformed with a luciferase reporter vector (0.12 μg) and 40 nM miR-106b mimic or negative control mimics. After transfection for 48 h, the dual luciferase reporter assay kit (Yuanping Hao Biotechnology Co., Ltd., China) was used for evaluation.

### Statistical analysis

All data are reported as means±SD. Comparisons between groups were performed with Student's *t*-test (two groups) or one-way analysis of variance followed by Fisher's LSD *post hoc* test (more than two groups). Statistical analysis was performed by Graphpad Prism 5 (Graphpad Software, USA). A P value less than 0.05 was considered to be significantly different.

## Results

### MiR-106b was down-regulated in HAECs

**Figure 1.**
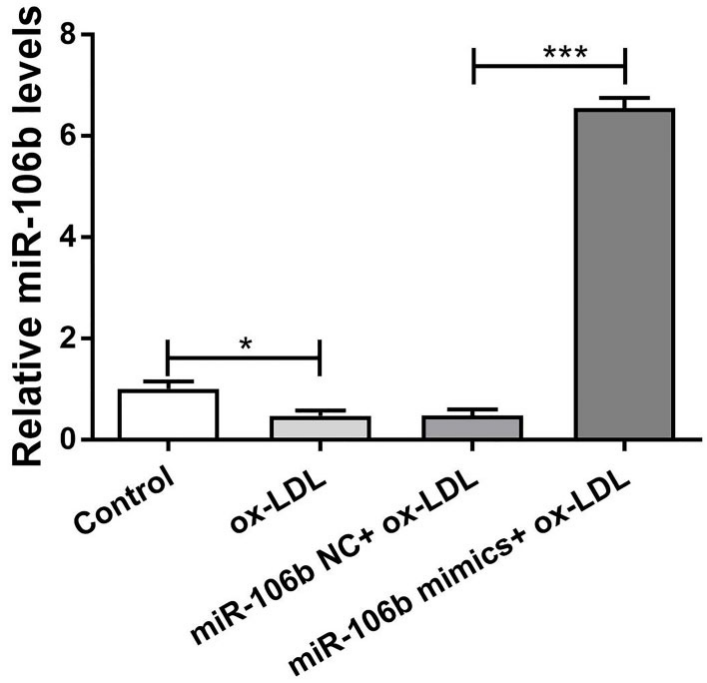
Expression level of microRNA-106b in oxidized-low-density lipoproteins (ox-LDL)-induced human aortic endothelial cells. Data are reported as means±SD. *P<0.05, ***P<0.001 (ANOVA). NC: negative control.

Compared with the control group, the expression of miR-106b in the ox-LDL group decreased significantly (P=0.014). Compared with the miR-106b NC+ox-LDL group, the expression of miR-106b in the mimics+ox-LDL group increased significantly (P<0.001, Figure 1).

### PTEN was the target gene of miR-106b

**Figure 2.**
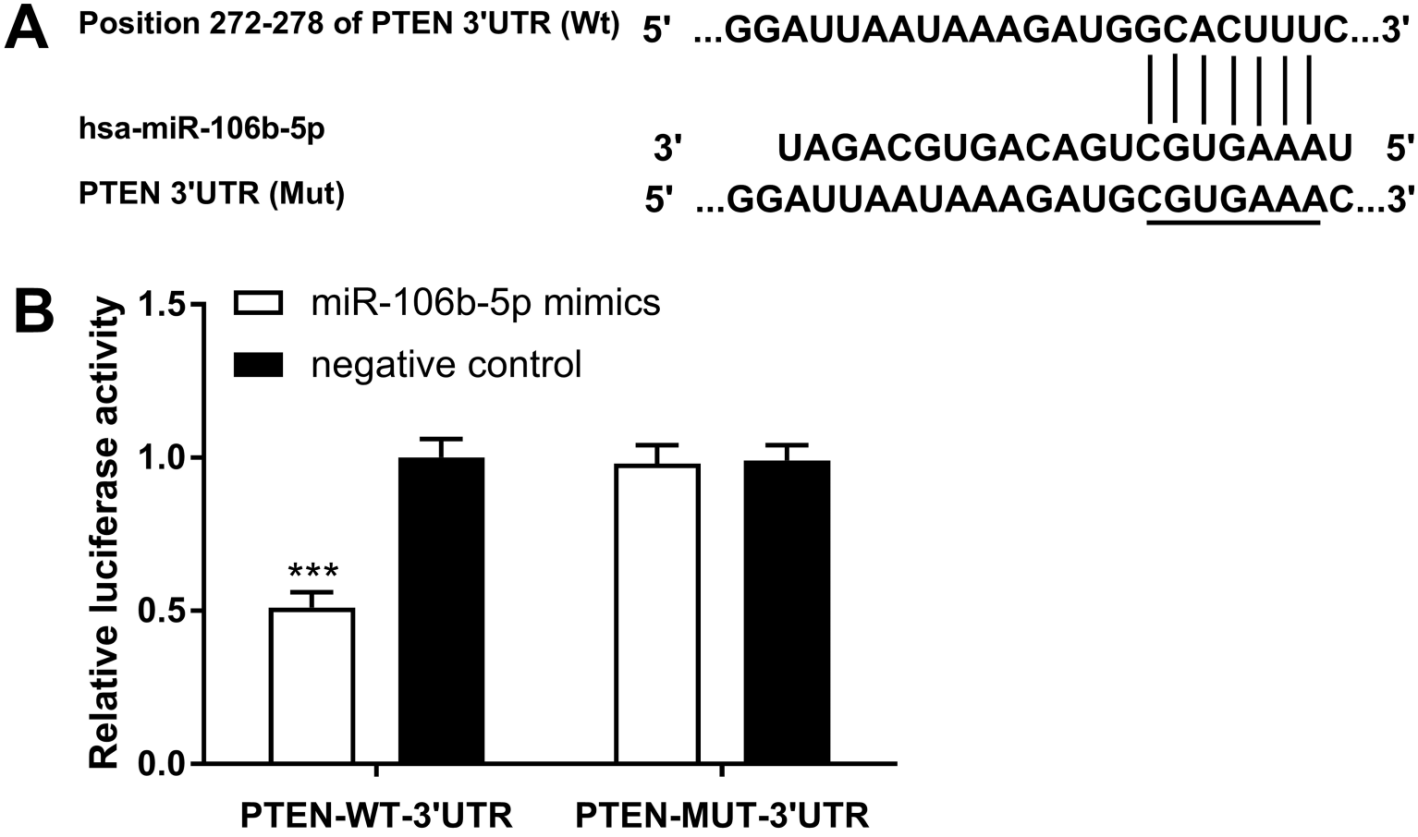
Relationship between PTEN and miR-106b. **A**, PTEN and miR-106b binding target sites predicted by Target Scan. **B**, Luciferase activity transfected with indicated luciferase reporters was determined using luciferase report assay. Data are reported as means±SD. ***P<0.001 *vs* miR-106b mimics and PTEN-MUT-3′UTR co-transfection (*t*-test).

The biological software Targetcsan predicted that the target gene of miR-106b was PTEN (Figure 2A). Moreover, the results of luciferase activity test showed that over-expression of miR-106b significantly decreased the luciferase activity of PTEN-WT-3′UTR, but did not inhibit the luciferase activity of PTEN-MUT-3′UTR (Figure 2B).

### MiR-106b inhibited the increase of PTEN in atherosclerosis

**Figure 3.**
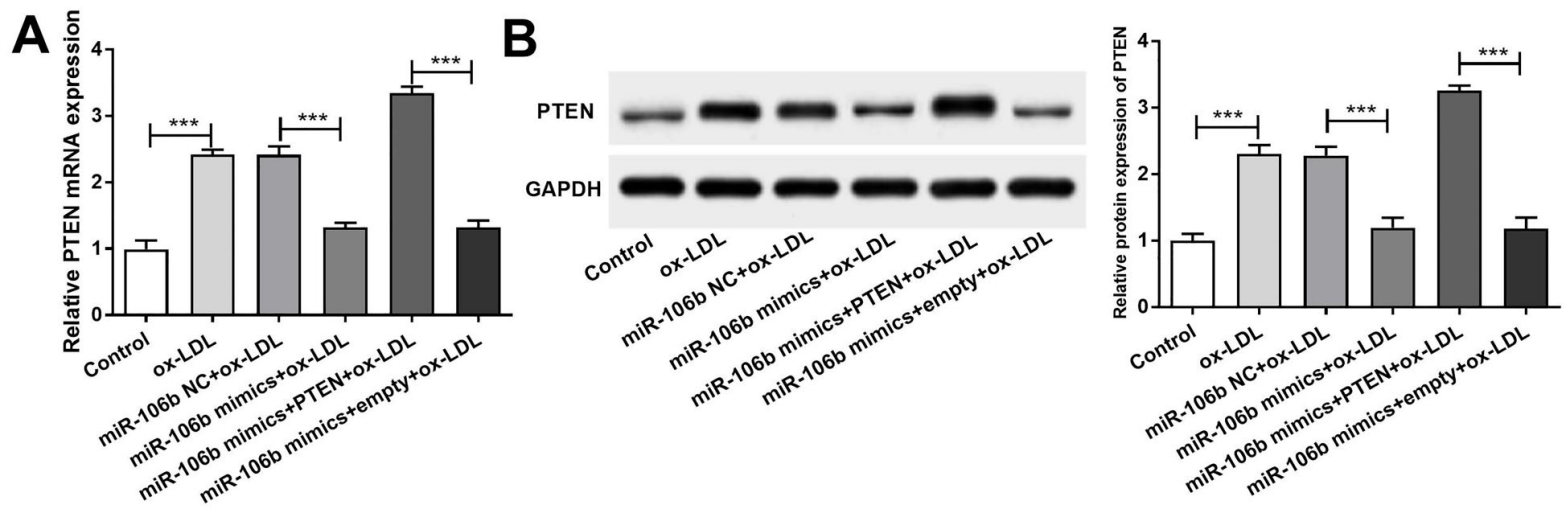
A, mRNA expression level of PTEN in the different groups of cells. **B**, Protein expression level of PTEN. Data are reported as means±SD. ***P<0.001 (ANOVA). ox-LDL: oxidized-low-density lipoproteins; NC: negative control.

The expression of PTEN mRNA and protein was detected by qRT-PCR (Figure 3A) and western blot (Figure 3B), respectively. The mRNA and protein levels of HAECs PTEN in the ox-LDL group were significantly higher than those in the control group (P<0.001). Meanwhile, the mRNA and protein levels of HAECs PTEN in the miR-106b mimics+ox-LDL group were significantly lower than those in the miR-106b NC+ox-LDL group (P<0.001). Moreover, the mRNA and protein levels of PTEN in the mir-106b mimics+PTEN+ox-LDL group were significantly higher than those in the mir-106b mimics+empty+ox-LDL group (P<0.001).

### Overexpression of miR-106b promoted proliferation and inhibited apoptosis of HAECs

**Figure 4.**
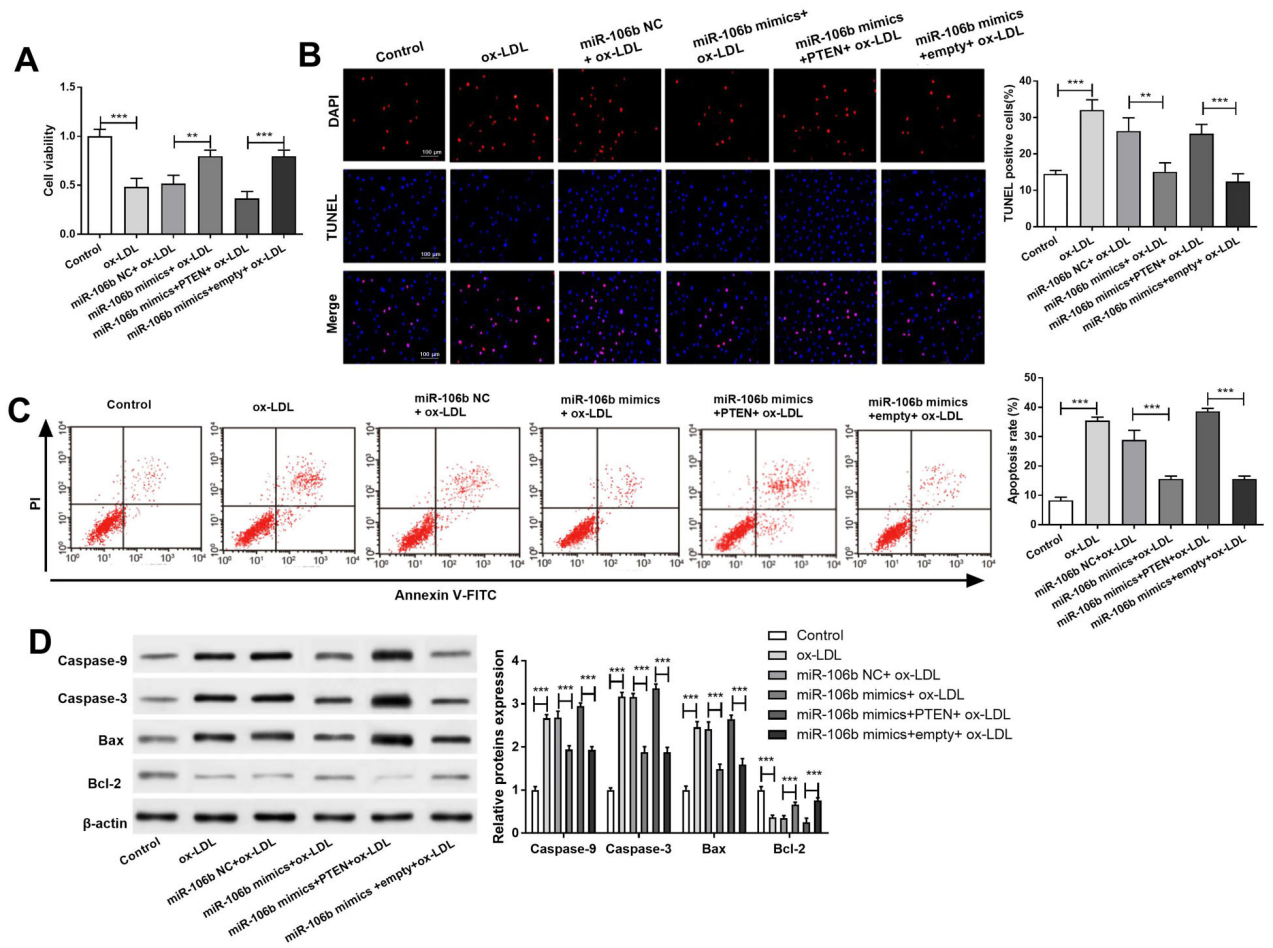
Overexpression of PTEN inhibited the anti-apoptotic effect of miR-106b. **A**, Proliferation of human aortic endothelial cells. **B**, Number of activity cells by TUNEL assay (×400, scale bar 100 μm). **C**, Apoptosis of human aortic endothelial cells. **D**, Expression of caspase-3, caspase-9, Bax, and Bcl-2 protein. Data are reported as means±SD. **P<0.01, ***P<0.001 (ANOVA). ox-LDL: oxidized-low-density lipoproteins; NC: negative control.

The proliferation of HAECs was detected by CCK-8 assay (Figure 4A). The activity of HAECs in the ox-LDL group was significantly lower than that in the control group (P<0.001). The activity of HAECs in the miR-106b mimics+ox-LDL group was significantly higher than that in the miR-106b NC+ox-LDL group (P<0.001). Meanwhile, the activity of HAECs in the miR-106b mimics+PTEN+ox-LDL group was significantly lower than that in the miR-106b mimics+empty+ox-LDL group (P<0.001).

To further investigate the relationship between HAECs activity and apoptosis, the DNA fragments were detected by TUNEL assay (Figure 4B). The number of TUNEL-positive cells in the ox-LDL group was significantly higher than that in the control group (P<0.001). Meanwhile, the number of TUNEL-positive cells in the miR-106b mimics+ox-LDL group was significantly lower than that in the miR-106b NC+ox-LDL group (P<0.01). Moreover, the number of TUNEL-positive cells in the miR-106b mimics+PTEN+ox-LDL group was significantly higher than that in the miR-106b+empty+ox-LDL group (P<0.001). Flow cytometry analysis showed that the apoptotic rate of HAECs in the ox-LDL group was significantly higher than that in the control group (P<0.001) (Figure 4C). The apoptotic rate of HAECs in the miR-106b mimics+ox-LDL group was significantly lower than that in the miR-106b NC+ox-LDL group (P<0.001). The apoptotic rate of HAECs in the miR-106b mimics+PTEN+ox-LDL group was significantly higher than that in the miR-106b mimics+empty+ox-LDL group (P<0.001). Western blot analysis showed that compared with the control group, the expressions of HAECs pro-apoptotic protein Bax, caspase-3, and caspase-9 in the ox-LDL group were significantly up-regulated (P<0.001), while the expressions of anti-apoptotic protein bcl-2 were significantly down-regulated (P<0.001) (Figure 4D). The expressions of HAECs pro-apoptotic protein Bax, caspase-3, and caspase-9 in the miR-106b mimics+ox-LDL group were significantly decreased (P<0.001), while the anti-apoptotic protein bcl-2 was significantly up-regulated (P<0.001). Furthermore, compared with miR-106b mimics+empty+ox-LDL group, Bax, caspase-3, and caspase-9 expression were significantly increased (P<0.001), and bcl-2 expression was significantly down-regulated (P<0.001) in the miR-106b mimics+PTEN+ox-LDL group. Taken together, these results suggested that overexpression of miR-106b in ox-LDL-treated AS could promote HAECs proliferation and inhibit HAECs apoptosis.

### MiR-106b targeted PTEN-activated PI3K/Akt signaling pathway in HAECs

**Figure 5.**
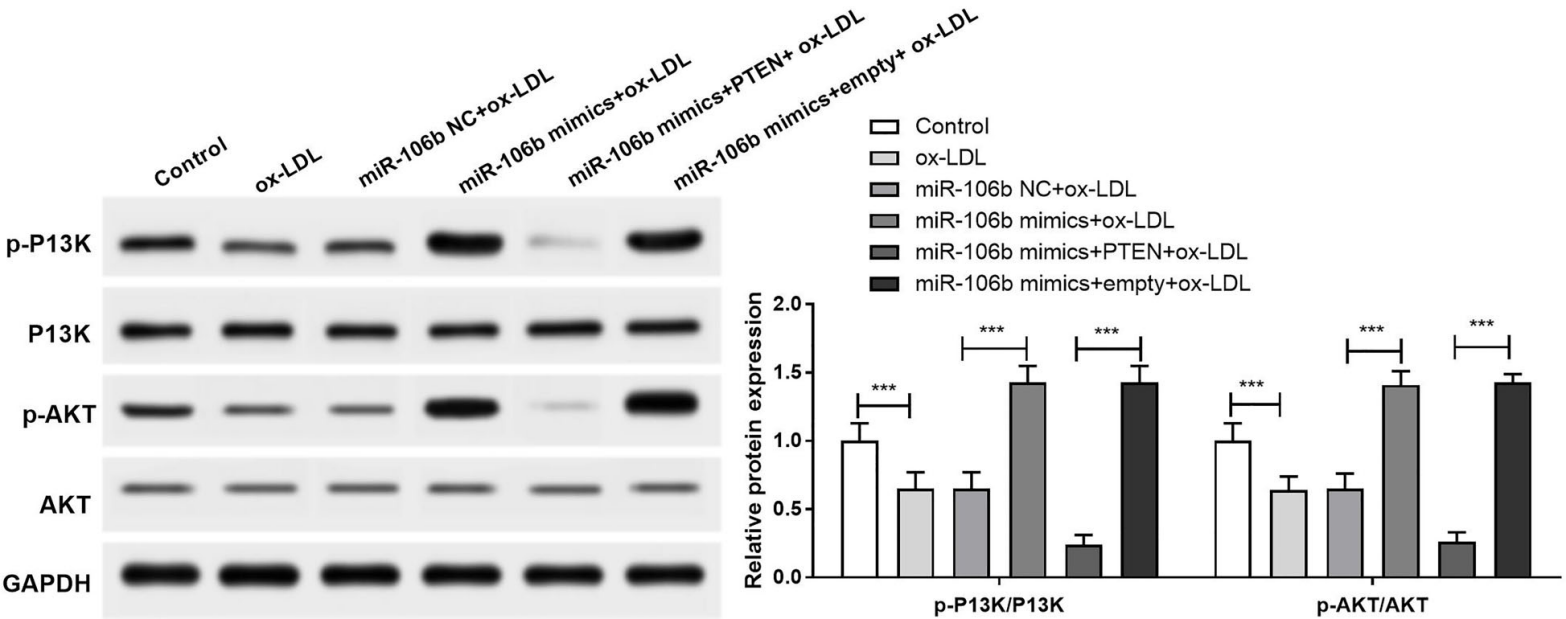
MiR-106b activated PI3K/AKT signaling pathway by down-regulating PTEN. Protein level of p-PI3K, total P13K, p-AKT, and total AKT was detected by western blot. Data are reported as means±SD. ***P<0.001 (ANOVA). ox-LDL: oxidized-low-density lipoproteins; NC: negative control.

Western blot assay showed that compared with the control group, p-P13K/PI3K and p-AKT/AKT expression in HAECs of the ox-LDL group were significantly down-regulated (P<0.001). Moreover, compared with the miR-106b NC+ox-LDL group, p-P13K/PI3K and p-AKT/AKT expression in the HAECs of the miR-106b mimics+ox-LDL group were significantly up-regulated (P<0.001). Furthermore, compared with the miR-106b mimics+empty+ox-LDL group, the expressions of p-P13K/PI3K and p-AKT/AKT in the miR-106b mimics+PTEN+ox-LDL group were significantly down-regulated (P<0.001) (Figure 5).

## Discussion

AS is an important cause of death and morbidity worldwide (23,24). Although miRNAs participate in ox-LDL-induced apoptosis of vascular endothelial cells (25), the detailed mechanism of miR-106b in AS is still unclear. In this study, ox-LDL-induced HAECs were used to simulate the pathological state of atherosclerotic endothelial cells. The results showed that miR-106b was down-expressed in AS endothelial cells, and overexpression of miR-106b promoted proliferation and inhibited apoptosis of AS endothelial cells. Importantly, miR-106b activated the PI3K/AKT pathway by down-regulating the level of PTEN.

Endothelial cell apoptosis is the first step in the pathogenesis of AS (26). As an anti-apoptotic modulator, the down-regulation of miR-106b can inhibit proliferation and migration of renal cancer cells and induce apoptosis (27,28). Li et al. (29) demonstrated that miR-106b-5p significantly enhances the expression level of Bcl-2 and reduces Bax expression. Importantly, members of the Bcl-2 gene family regulate programmed cell apoptosis by controlling intracellular signaling promoting apoptosis and anti-apoptosis (30). However, a pervious study shows that overexpression of Bax accelerates apoptosis induced by cytokine deprivation in cell lines (31). Skała et al. (32) indicated that the high expression of pro-apoptotic Bax protein and low expression of anti-apoptotic Bcl-2 protein are beneficial to inhibit cell proliferation and promote apoptosis. More importantly, Bcl-2 protein also activates the downstream caspase cascade by regulating mitochondrial extracorporeal membrane permeabilization to perform apoptosis (33). Down-regulation of Bcl-2, pro-caspase-3, and pro-caspase-9 can accelerate the proliferation of HAECs and promote endothelial cell apoptosis, thereby promoting the formation of AS (34). In fact, miR-106a mediates the caspase-3 pathway (35). The apoptotic rate and caspsse-3 activity are significantly increased when the expression of miR-106b is inhibited (35). miR-106b prevents the apoptosis of endothelial cells in AS by blocking the activation of caspase-3 (36). In this study, the expression of Bax, caspase-3, and caspase-9 in cells transfected with miR-106b was significantly decreased, while Bcl-2 was significantly up-regulated. Thus, we speculated that the overexpression of miR-106b in ox-LDL-induced AS inhibited endothelial cell apoptosis.

A previous study shows that the inhibition of miR-106b expression enhances the expression of PTEN (37). The up-regulation of PETN can induce endothelial cell dysfunction by attenuating the effectiveness and signaling of various angiogenic pathways in endothelial cells, which are involved in thrombosis of arteriovenous grafts (38). Moreover, overexpression of PTEN inhibits cell proliferation and increases the probability of apoptosis by inhibiting Bcl-2 and promoting caspase-3 (39). As the downstream of PTEN, p-Akt is positively regulated by the overexpression of miR-106b (40). A previous study indicates that the inhibition of miR-106b expression enhances PTEN level, but inhibits the activity of downstream PI3K/AKT pathway (37). Meanwhile, inhibition of the PTEN expression level in vascular endothelial cells by miR-106b-5p prevents apoptosis of endothelial cells in AS (36). In the current study, the Targetcsan prediction and luciferase activity test showed that PTEN was a direct target of miR-106b. Meanwhile, western blot assay showed that overexpression of miR-106b inhibited endothelial cell apoptosis via the PTEN/P13K/AKT signaling pathway. Thus, we speculated that miR-106b might activate the PI3K/AKT pathway by down-regulating the expression of PTEN in ox-LDL induced HAECs.

In conclusion, overexpression of miR-106b in ox-LDL-induced AS inhibited endothelial cell apoptosis. Furthermore, miR-106b might activate the PI3K/AKT pathway by down-regulating the expression of PTEN in ox-LDL-induced HAECs. However, there are some limitations in this study including lack of clinical verification. Thus, future research is still needed.
